# Post-Operative Analgesics Score, Another Tool in the Quest for a Better Pain Management

**DOI:** 10.1007/s00268-023-07101-6

**Published:** 2023-07-03

**Authors:** Ruben Peralta, Gustav Frans Strandvik

**Affiliations:** 1 Department of Surgery, Trauma Surgery, Hamad General Hospital & Hamad Medical Corporation, Doha, Qatar; 2 Department of Surgery, Universidad Nacional Pedro Henriquez Urena, School of Medicine, Santo Domingo, Dominican Republic

This edition of the World Journal of Surgery addresses an issue of global humanitarian, economic, and even political importance [1]. It has been termed the ‘fifth vital sign’ and is responsible for a significant economic burden to both individuals and society [2].

Although acute pain has obvious implications in terms of resource utilization and humane treatment, it is chronic pain resulting from surgical procedures that has the greater impact on quality of life and economic activity. A significant proportion of the harm associated with chronic pain is its’ treatment; the opioid epidemic in the USA and worldwide illustrates the dangers of managing chronic pain with medications designed to relieve acute pain [3]. No standardized therapy for chronic post-surgical pain (CPSP) exists; preventative strategies thus deserve focused attention.

This holds true especially in inguinal hernia surgery, an area where the confluence of three nerves and their surgical handling are associated with a significant incidence of debilitating chronic post-inguinal surgery pain (CPIP) [4].

Preventing CPIP remains a challenge. Experienced surgeons may suspect that an individual, highly anxious patient may be at higher risk of CPIP, but clinical practice often confounds even the most astute clinician. Identifying at-risk patients is thus a key component in managing this entity. Understanding that a hot, dry summer increases the risk of forest fires contributes to enhanced vigilance. Preventing the spark that *starts* the fire becomes the focus of prophylactic interventions, and rescue plans are readily accessible, with all relevant parties on high alert.

Surprisingly, pre-emptive analgesic strategies aimed at reducing chronic post-surgical pain, and CPIP, have shown inconsistent results [5]. Regional anesthetic techniques continue to hold out the tantalizing prospect of being the universal panacea. However, definitive evidence is not strong enough to make this the de facto approach in all patients.

Peri-operative risk factors for CPIP have been enumerated and validated in small series. Model predictive ability remains too weak to provide consistent for reliable use; none the less open repair of recurrent hernias in young females with high pre-operative pain scores seems to confer the highest risk [6]. Intra-operative approaches have appeared to have limited influence on CPIP. Importantly, early high post-operative pain has consistently been identified as risk factor.

Generic pain scores have existed for decades, the most validated include the numeric rating scale (NRS). Obvious shortcomings include the absence of dynamic measures and the ‘single snapshot’ nature of the pain evaluation. Consequently, specific pain assessment has been developed for specific pain-inducing phenomena. Examples include the ‘PIC’ Score, a focused, inverse pain assessment tool for rib fracture patients’. Pain on movement, cough strength and inspiratory effort—all are linked to the need for enhanced analgesic interventions.

Clear associations between the severity of post-operative pain and the development of chronic post-surgical pain have been demonstrated.

Person-centered, precision medicine-based approaches to CPIP prevention are awaited; genotype mapping may soon help identify patients at risk of CPSP. However, phenotypic identification of particular patient patterns in the PACU will remain a key tool.

Widder and colleagues therefore need to be congratulated for developing the first acute post inguinal hernia repair pain score. This may provide a small part of the puzzle, and allow aggressive, targeted interventions immediately post herniorrhaphy. We encourage the launch of a prospective and multi-institutional study to validate the scoring tool described.

Surgical techniques have been developed to address this common clinical condition; three main procedures are recommended and used frequently, the open surgical approach (Lichtenstein) and two minimal invasive techniques: extraperitoneal (TEP) and Transperitoneal (TAAP) inguinal hernia repair, but unfortunately previous studies have demonstrated mixed results, in relation with high risk of recurrences, post-operative complications and CPIP. It is important to take in consideration as a clinician, that post- operative pain that develop after a hernia repair procedure, will cause a significant number of patients to suffer CPIP long-term, and even permanently.

Outstanding challenges therefore remain; including what the most effective management of acute pain post-surgery should be.
